# Three-Year Results of Management of Adult-Onset Coats' Disease by Possibly Targeting Placental Growth Factor

**DOI:** 10.7759/cureus.10652

**Published:** 2020-09-25

**Authors:** Khalid Alsaggaf, Motaz Jalloun, Waiel Alkhotani, Mohammed Albeedh

**Affiliations:** 1 Ophthalmology, National Guard Health Affairs, Jeddah, SAU; 2 Ophthalmology, Saggaf Eye Center, Jeddah, SAU

**Keywords:** coats' disease, optical coherence tomography, retinal telangiectasia, retinal exudative vasculopathy, subretinal exudates, nonhereditary ocular disease, adult-onset coats' disease, aflibercept

## Abstract

We report long-term results of treatment with intravitreal injection of aflibercept in a newly diagnosed case of adult-onset Coats' disease. A 40-year-old Philipino male was referred to our Eye Center complaining of vision reduction for the past three months in his right eye. Examination revealed visual acuity of 0.1 in the affected right eye with normal vision of 1.0 in his left eye. Fundal examination of the right eye showed temporal retinal telangiectasia with massive exudation reaching the macula. The left fundal examination was normal. The patient’s optical coherence tomography (OCT) demonstrated massive exudative thickening. The patient was managed with one intravitreal injection of aflibercept followed by focal laser, with successful anatomical and subjective outcomes that were maintained after three years. Aflibercept's profile targeting both vascular endothelial growth factor (VEGF) and placental growth factor seems to show favorable outcomes when compared to other purely anti-VEGF treatment options. We therefore believe that aflibercept is an excellent adjunctive therapy in cases of adult-onset Coats’ disease with macular edema. To our knowledge, this is the first case treated with laser and intravitreal aflibercept with a long three-year follow-up suggesting the significant role of aflibercept in vascular permeability and supporting its use as a valuable therapeutic option for Coats’ disease.

## Introduction

Coat’s disease is a rare non-hereditary retinal telangiectasia or aneurysm that might lead to retinal exudation. In 1908, George Coats was the first to describe the disease [1]. Adult-onset Coats’ disease makes up a small percentage of patients with Coats’ disease, with an average age of onset of approximately 50 years. Patients with adult-onset Coats’ disease characteristically have more retinal hemorrhages adjacent to the telangiectasia and have a better visual prognosis due to more localized pathology. This case report is the first to our knowledge to find that intravitreal aflibercept accompanied by focal laser is highly efficacious in the long term for resolving intraretinal and subretinal macular edema and maintain visual vision in adult-onset Coats’ disease.

## Case presentation

**Figure 1:**
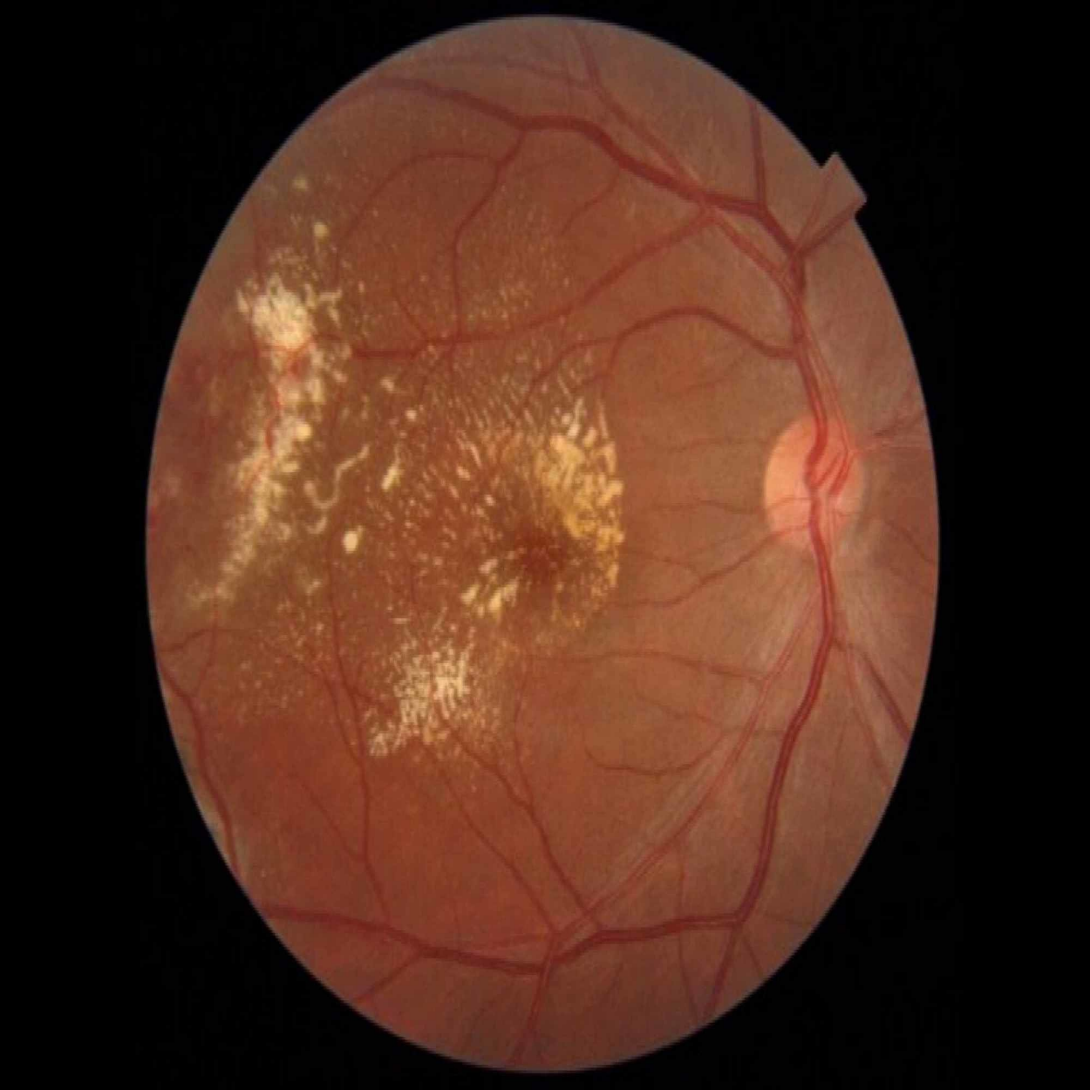
Fundoscopy showing temporal retinal telangiectasia with massive exudation reaching the macula in the patient's right eye.

**Figure 2:**
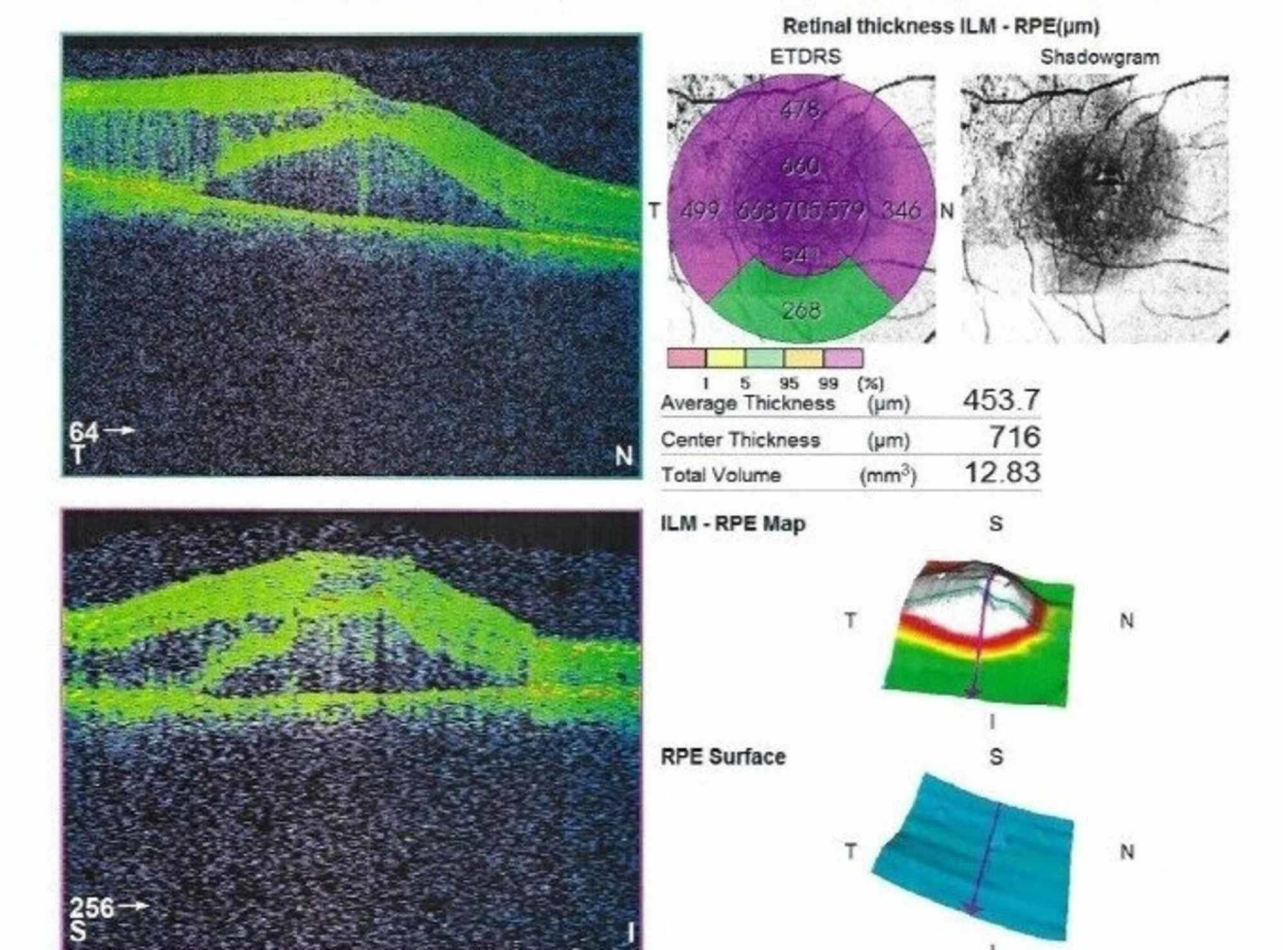
OCT showing central retinal thickness of 716 µm in the patient's right eye pre-management. OCT, optical coherence tomography

**Figure 3:**
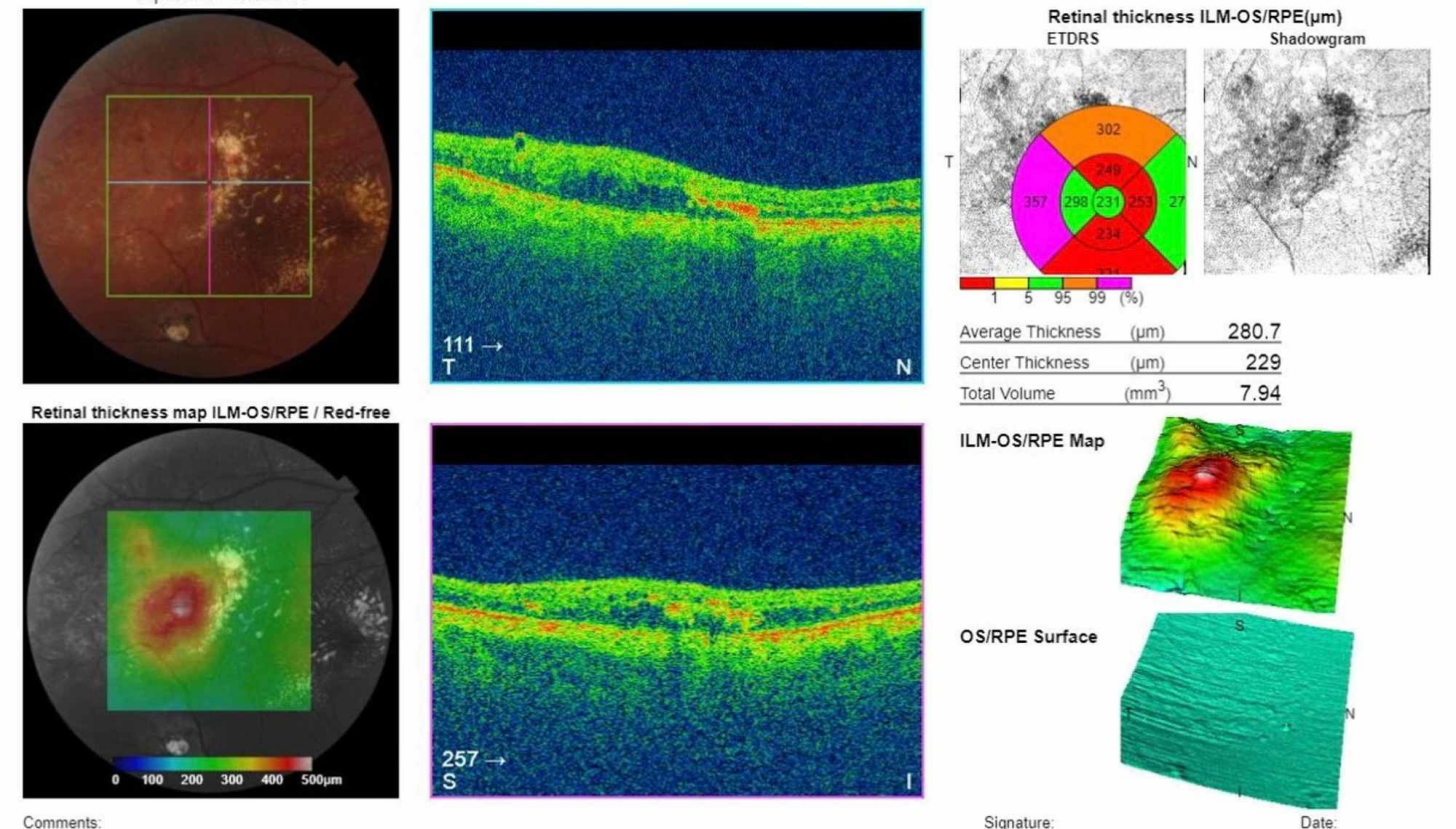
OCT showing central retinal thickness in the patient's right eye of 229 µm post-management. OCT, optical coherence tomography

A 40-year-old Philipino male was referred to our Eye Center complaining of vision reduction persisting throughout the past three months in his right eye. The patient had a history of allergic hypersensitivity to fluorescein; his past medical history and ocular history were otherwise unremarkable. The patient did not have systemic hypertension, diabetes mellitus, or hypercholesterolemia. He did not have a history of retinal detachment, exposure to radiation, episodes of intraocular inflammation, tapetoretinal degeneration, or vascular occlusion. His family history was negative, and he was not on any medications or a user of illicit drugs. Best-corrected visual acuity (BCVA) was 0.1 OD and 1.0 OS. The patient was orthophoric and had normal intraocular pressure in both eyes. Examination of the anterior segment in both eyes revealed clear corneas with deep anterior chambers and clear crystalline lenses. There was no rubeosis iridis. Dilated fundoscopy of the right eye revealed a clear vitreous with temporal retinal telangiectasia and massive exudation reaching the macula in the right eye (Figure 1). There were no signs of retinal degeneration or detachment. Dilated fundoscopy revealed normal vasculature in the left eye. Dilated optical coherence tomography (OCT) demonstrated intraretinal and subretinal macular fluid with a central retinal thickness of 716 µm (Figure 2). Fluorescein angiography could not be performed due to the patient’s allergic status. Ultrasound B scan was unnecessary as there was no bullous retinal detachment with exudation, which may have resulted in a diagnostic dilemma. A work-up demonstrated normal CBC (complete blood count), PT (prothrombin time), aPTT (activated partial thromboplastin time), INR (international normalized ratio), HbA1C (hemoglobin A1c), and lipid levels. Coagulopathy studies revealed normal levels of homocysteine, protein S, protein C, and no factor V Leiden mutations. Thus, based on the aforementioned findings, the diagnosis of adult-onset coat’s disease (stage 2B) was made. The patient was managed with one aflibercept injection, and two weeks later, he was examined with a marked clinical reduction in the macular edema, whereby laser is amenable at this stage. Then, the patient received one session of argon-laser photocoagulation targeting leaking blood vessels as a chronic management. Four weeks later, the patient demonstrated excellent resolution of the macular edema, resulting in an outstanding improvement in the visual acuity in the right eye from 0.1 to 0.7 with a significant reduction in the central retinal thickness from 716 µm to 229 µm (Figure 3). The patient presented to the clinic for three monthly follow-ups with thorough ocular examinations at each visit. At each follow-up, visual acuity, intraocular pressure, neovascular changes in the iris or angle, and fundus evaluations for disease progression were carried out. His follow-ups demonstrated consistent stability in his BCVA and OCT macular status, and after three years, it revealed that visual acuity was maintained at 0.7 with no evidence of recurrent macular edema.

## Discussion

Coat’s disease is a rare non-hereditary retinal telangiectasia or aneurysm that might lead to retinal exudation. In 1908, George Coats was the first to ever describe the disease [1]. Coat’s disease in adulthood is characterized by the presence of retinal telangiectasia, aneurysms, and abnormal dilatation of retinal blood vessels. Adult-onset Coats’ disease is characterized by a progressive exudative retinal vasculature that might lead to partial retinal detachment or, eventually, total retinal detachment in advanced cases [2]. Dimness and decreased vision are among the most common presenting symptoms in adult-onset Coat’s disease, accounting for 83% of case presentations. The exact etiology of the disease is unknown, with multiple studies suggesting that hypertension, diabetes mellitus, and hypercholesterolemia are often present in patients with adult-onset Coat’s disease [3]. Coats' disease is diagnosed clinically, and the tools that help reaching the diagnosis include fundoscopy, OCT, and fluorescein angiography. Late onset of the disease is associated with slow progression and better prognosis. Different treatment options such as laser photocoagulation, cryotherapy, anti-vascular endothelial growth factor (VEGF) injections, surgical treatment for exudative retinal detachment, and enucleation (in severe cases that are non-responsive to the previous options) are available for the resolution of the disease [4]. The approach toward disease management in this case relied on the traditional modality of the management of Coats’ disease: laser photocoagulation. However, a study published in 2014 that had concluded that the increased severity of Coats’ disease was significantly associated with increased levels of VEGF was considered [5]. Another study published in June 2019 highlighting the importance of using anti-VEGF in adjunction with the traditional modality involving laser photocoagulation was also considered [4]. Our approach, which resulted in favorable outcomes, supports and demonstrates the validity of the previous studies. In selecting the most effective anti-VEGF injection to be used, multiple studies were selected for further review. One case of a 17-year-old female diagnosed with Coat’s disease, initially treated with bevacizumab injection, reported poor outcomes. However, upon substituting the injection with aflibercept instead, resolution of the refractory edema and an overall improvement in visual acuity were reported in the same patient [6]. Another case reported a 14-year-old boy diagnosed with Coats’ disease who received six ranibizumab injections in addition to three laser photocoagulation sessions. In this patient, the edema persisted with poor improvement of visual acuity from 0.1 to 0.2 [7]. Following four aflibercept injections and two additional laser photocoagulations, the patient’s visual acuity improved from 0.2 into 0.5, with good reported foveal slope and no recurrence at 12-month follow-up [7]. It was consequently determined that cases within the literature have determined better efficacy of aflibercept over other anti-VEGF injections, such as bevacizumab and ranibizumab [6,7]. With proven efficacy, there appears to be little understanding within the literature as to why aflibercept may be the most effective in the management of Coats’ disease. A 2017 study quantifying growth factor levels in the aqueous humour of macular telangiectasia type 1 (MacTel 1) patients using a multiarray immunoassay showed that compared with control subjects, the profile of angiogenic factors in MacTel 1 eyes revealed significantly higher levels of placental growth factor (P = 0.029). The authors suggested that the eyes’ clinical response to aflibercept coupled to the angiogenic profile of MacTel 1 eyes supported the implication of the placental growth factor/Flt-1 pathway in MacTel 1. As MacTel 1 appears to share a similar pathogenetic development with Lebers’ milliary aneurysm and Coats’ syndrome as telangeictatic and aneurysmal retinal diseases, the authors postulate that the better efficacy of aflibercept compared to other anti-VEGF injections may be due to its anti-placental growth factor activity [8]. Comparing effects between different anti-VEGF modalities in such a rare disease such as adult-onset Coat's disease requires studies on a larger scale, which we know is not possible in such rare cases.

## Conclusions

Laser photocoagulation and cryotherapy remain gold standard modalities, but growing evidence suggests a role of anti-VEGF modalities. Aflibercept’s profile targeting both VEGF and placental growth factor seems to show favorable long-term outcomes when compared to other purely anti-VEGF treatment option. We therefore believe that aflibercept is an excellent adjunct in cases of adult-onset Coat's disease with macular edema.
